# Cell Type Specific Expression of Toll-Like Receptors in Human Brains and Implications in Alzheimer's Disease

**DOI:** 10.1155/2019/7420189

**Published:** 2019-07-18

**Authors:** Henriette R. Frederiksen, Henriette Haukedal, Kristine Freude

**Affiliations:** Group of Stem Cells and Modeling of Neurodegeneration, Department of Veterinary and Animal Sciences, Faculty of Health and Medical Sciences, University of Copenhagen, Grønnegardsvej 7, 1870C Frederiksberg, Denmark

## Abstract

Toll-like receptors mediate important cellular immune responses upon activation* via* various pathogenic stimuli such as bacterial or viral components. The activation and subsequent secretion of cytokines and proinflammatory factors occurs in the whole body including the brain. The subsequent inflammatory response is crucial for the immune system to clear the pathogen(s) from the body* via* the innate and adaptive immune response. Within the brain, astrocytes, neurons, microglia, and oligodendrocytes all bear unique compositions of Toll-like receptors. Besides pathogens, cellular damage and abnormally folded protein aggregates, such as tau and Amyloid beta peptides, have been shown to activate Toll-like receptors in neurodegenerative diseases such as Alzheimer's disease. This review provides an overview of the different cell type-specific Toll-like receptors of the human brain, their activation mode, and subsequent cellular response, as well as their activation in Alzheimer's disease. Finally, we critically evaluate the therapeutic potential of targeting Toll-like receptors for treatment of Alzheimer's disease as well as discussing the limitation of mouse models in understanding Toll-like receptor function in general and in Alzheimer's disease.

## 1. Introduction

For many years it was believed that the brain did not possess an immune system, due to its isolation* via* the blood-brain barrier (BBB). The BBB represents a physical and anatomical barrier regulating uptake and release of molecules into the nervous tissue. In this manner, the brain is protected from the rest of the body, ensuring homeostasis of the cellular environment, which is essential for proper neuronal function [1]. The barrier limits entry of undesirable and/or toxic molecules and provides a means of removal of toxic substances produced in the brain. On the other hand, the BBB hinders delivery to the brain of nutrients and growth factors required for proper metabolism and nervous function [2]. Over the past decades it has become clear that the BBB is not as restrictive a barrier as previously assumed and that the brain is equipped with an innate immune system including specialized cells mediating such immune responses [3]. Research within, for example, the arena of Alzheimer's disease (AD) has revealed that neuroinflammation plays an important role in the disease mechanism and the brain's efforts to relieve the burden of amyloid plaques. Other well-documented examples of brain innate immune system activation involve traumatic brain injuries [4]. The core cells of the brain's innate immune system comprise microglia and astrocytes, which are gaining increasing attention as regards their involvement in disease development and progression. Amongst the neurodegenerative disorders, AD is considered to be the most common, affecting millions of people worldwide, with no curative treatment currently available. Historically, most researchers have focused their efforts on Amyloid beta (A*β*) plaques and neurofibrillary tangles (NFT), which together form the major histological hallmarks identified in postmortem AD patient brains [5]. Surprisingly and unfortunately, all therapeutic efforts to lower A*β* production and plaque load, manifesting in reversal of pathological hallmarks in AD mouse models, have to date shown negligible effects in human clinical trials. Consequently, the focus of research efforts that aimed at understanding AD pathology for therapeutic purposes has shifted and the inflammatory component of AD has taken a central stage, with immunotherapy being a potentially promising approach. Although a number of antibodies targeting A*β* have to some degree been shown effective in reducing the A*β* burden in animal models, the overall clinical trials have not shown the same results [6]. The lack of positive results could however be due to the fact that treatment was started too late, and inflammation remains as a promising target in AD therapeutics. Neuroinflammation can be considered as a third hallmark in AD, highlighting the role of nonneuronal cell types and showing AD to be a multicellular pathogenesis. Increased activation of microglia and astrocytes has been identified in AD, manifesting in release of proinflammatory cytokines and neurotoxic mediators such as tumor necrosis factor-alpha (TNF-*α*), interleukin-1*β* (IL-1*β*), IL-6, IL-12, and IL-18, together with upregulated production of neurotoxic mediators including proteolytic enzymes, complement factors, nitric oxide (NO), and reactive oxygen species (ROS) [7]. These cell populations detect and respond to various stimuli such as pathogens and protein aggregates, as seen in AD, through the activation of various cell surface receptors. An important group of such receptors comprises the Toll-like receptors (TLRs). An increasing body of evidence supports an association between these receptors and various neurodegenerative disorders. TLRs not only are expressed by microglia and astrocytes, but have also been identified on neurons and oligodendrocytes in the brain [8]. However, the main focus of this review will be the immune cells of the brain, namely, microglia and astrocytes. We will provide a general introduction to AD and neuroinflammation, followed by a review and discussion of the involvement of TLRs in disease pathology and the inflammatory response.

## 2. Alzheimer's Disease

AD is a neurodegenerative disorder characterized by a progressive decline in memory and cognitive abilities. AD is the most common form of dementia, accounting for around 60-80% of all dementia cases, and is the leading cause of disability in the elderly population, affecting approximately 50 million people worldwide [9]. With age being the most recognized risk factor for developing AD, the disease is rapidly becoming an increasing health challenge with a continuously aging population and no curative treatments currently available. AD can be characterized as either familial (fAD) in which mutations in amyloid precursor protein (*APP*), presenilin 1 (*PSEN1*), or presenilin 2 (*PSEN2*) are causative of the disease, or sporadic (sAD) with no apparent heritability. The latter accounts for the majority of all cases and is associated with genetic risk factors that in combination with adverse environmental factors confer a certain risk of developing the disease [10]. fAD and sAD share the same pathophysiology. Primarily neurons degenerate and lose their function, eventually resulting in severe brain atrophy. The AD brain is characterized by two major neuropathological hallmarks, namely, extracellular deposits of A*β* in the form of senile plaques and intracellular formation of NFTs caused by tau hyperphosphorylation [5]. These hallmarks are mainly restricted to neuronal pathology. However, emerging evidence implies that immunological processes occur alongside the degenerating neurons, indicating potential roles of microglia and astrocytes, hence, neuroinflammation as a contributor to AD development and progression. Glial activation has been identified in patients with AD [11] and elevated levels of cytokines, chemokines, and complement factors in both the brain and cerebrospinal fluid (CSF) have been observed in these patients, indicating ongoing neuroinflammation. However, the results of studies investigating the levels of cytokines in the CSF of AD patients are controversial, and the time point of sampling and stage of disease have proved to be an important factor in such studies [12]. Distinct stages of microglial activation have been suggested to occur during the course of disease. In early phases of AD, activated microglia migrate towards A*β* deposits and clear them by phagocytosis, thus providing a protective effect of increased microglial activation. However, failure to adapt to chronic A*β* deposition results in incorrectly modulated activation levels and possibly leads to a shift towards a dysfunctional or neurotoxic microglial phenotype in later AD stages [13, 14]. However, whether neuroinflammation is in fact a cause or consequence of neurodegeneration remains to be elucidated: whether the inflammatory response precedes tau and A*β* aggregation is the focus of ongoing debate.

### 2.1. Neuroinflammation in AD

It has become clear that neuroinflammation is an important contributor to the complex pathology of AD. Genome-wide association studies have revealed a number of genes to be associated with increased risk of AD, with many of these being expressed by immune cells, indicating a multicellular pathogenesis and a primary role of neuroinflammation in AD aetiology [15]. A well-established example is the gene encoding the* triggering receptor expressed on myeloid cells 2* (*TREM2*). TREM2, which is highly expressed by microglia, acts as a regulator of phagocytosis and cytokine production; variants within this gene have been observed in AD patients [16]. Other identified genetic risk factors include CLU, CR1, and CD33, all associated with the innate immune system [17], thus further strengthening a link between inflammation and AD. Aggregation of proteins has been observed to activate microglia and astrocytes, and A*β* deposition in AD can trigger an innate immune response. Microglia respond to A*β* which initiates migration to the plaques and phagocytosis of A*β*, alongside release of proinflammatory cytokines. Accumulation of activated microglia has been observed around A*β* plaques in both mouse models and postmortem AD brains [18, 19]. However, several animal AD models have shown that prolonged activation decreases microglial efficiency in terms of A*β* clearance, while the production of neurotoxic cytokines sustains. The compromised A*β* clearance and persistent release of proinflammatory mediators in turn damage nearby neurons further promoting neurodegeneration, accelerating disease progression [20]. A*β* can react with microglial surface receptors and stimulate either the NF-*κ*B-dependent pathway or activation of mitogen-activated protein kinase (MAPK) pathways, inducing proinflammatory gene expression. A*β* has also been documented to induce NADPH-oxidase-mediated ROS production in microglia, resulting in increased neurotoxicity and neurodegeneration [21]. Although some of the inflammatory mechanisms involved in AD are understood, there is still much debate as to whether neuroinflammation is causative for AD, as the identification of genetic risk factors associated with the innate immune system might imply, or if it is a consequence of other AD pathologies such as A*β* accumulation. Precise pathways and other mechanisms of microglial response in disease thus remain to be elucidated.

As microglia are the resident immune cells of the central nervous system (CNS), dysfunction in this cell population is gaining increased attention in terms of the neuroinflammatory response in AD. Normally microglia exist in a “resting” state, fulfilling such duties as synaptic pruning, to ensure proper neuronal connectivity. In addition, they play a role in modulating cognitive functions, such as learning and memory, and maintain brain homeostasis by secreting neurotrophic factors that promote differentiation and survival of neurons and by scavenging and removing defective neurons by inducing neuronal death [22]. As such, microglia perform “immune surveillance” in the brain: they become activated in the presence of various stimuli, such as pathogens or tissue damage, to eliminate the potential threat. Traditionally, activation of microglia has been categorized as having either a proinflammatory, toxic state, or an alternative, protective state. In response to stimuli, these cells have been suggested to change their phenotype either into the classical “M1” state, with secretion of proinflammatory cytokines such as TNF-*α*, IL-6, and IL-1*β* and cytotoxic factors such as NO and ROS, or to the alternative “M2” state, with secretion of anti-inflammatory cytokines such as TGF-*β* and IL-10 and neurotrophic factors such as BDNF and GDNF. The latter is thus integral to the downregulation of inflammation to restore CNS homeostasis [23]. This classification can be limiting as it only represents two opposite sides of the activation continuum. Accumulating evidence suggests that the activation profile of microglia is multidimensional, indicating the need of new terminology based on emerging data within transcriptomics, gene expression, and proteomic analyses [24]. However, the traditional terms will be further used in this review, to clearly distinguish between a neurotoxic and neuroprotective microglial state and their potential beneficial and detrimental effects in neurodegenerative disorders. Many of the studies involving microglial activation have been performed in animal models and have yet to be confirmed in humans. Such studies give an important insight into the possible mechanisms of neuroinflammation but need to be proven in human brain studies to fully understand the human disease pathophysiology.

In addition to the release of inflammatory mediators, activated microglia facilitate the crucial process of phagocytosis, to clear pathogens, debris, or protein aggregates, maintaining the brain homeostasis.

Accumulation of activated microglia has been detected in tissue from AD brains, with this activation being particularly evident around A*β* plaques, indicating that microglia can be activated by A*β*. These findings go hand-in-hand with increased proinflammatory factors in these patients, which might exert detrimental effects on surrounding neurons, exacerbating disease progression [18]. Conversely, activated microglia can, as shown in transgenic mouse models, to some extent, clear the accumulating A*β* oligomers through phagocytosis, providing beneficial effects in AD pathogenesis [25]. The role of microglia in AD is thus very complex, with a potential beneficial activation in early disease stages and detrimental activation in late disease stages. It has been suggested that dysfunction in these cells promotes the neurotoxic effects and diminishes the neuroprotective effects of microglia. Targeting the regulation of microglial activation might thus serve as a potential avenue to pursue in the development of AD therapeutics. However, strategies for targeting microglia and neuroinflammation would have to be intricately tailored to the stage of the disease, promoting the beneficial neuroprotective activation in early stages and suppressing the neurotoxic effects in later stages of the disease course [26].

**Figure 1:**
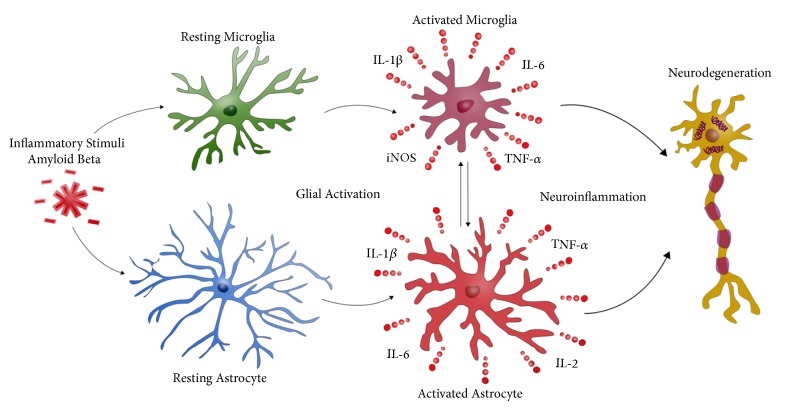
The potential role of neuroinflammation in Alzheimer's disease. Chronic exposure to inflammatory stimuli such as amyloid beta (A*β*) stimulates neurotoxic activation of microglia and astrocytes, triggering the release of proinflammatory cytokines and reactive oxygen species, promoting degeneration of neurons.

Alongside microglia, astrocytes are also currently attracting increased attention for their potential role in AD progression and likewise converge around A*β* plaques in the brains of AD patients [7]. Astrocytes are the resident cells of the CNS that play key roles in maintaining brain homeostasis, in processes such as uptake and recycling of neurotransmitters, release of gliotransmitters and nutrients, and regulation of synaptic activity and inflammation [27]. Astrocytes can release transmitters such as glutamate through calcium-dependent exocytosis. However, astrocytes can also take up glutamate* via* plasma membrane transporters, thereby serving important functions in both neuronal and glial communication and in glutamate balance, with potential impacts on excitotoxicity [28]. Astrocytes also closely interact with synapses and play a role in synapse formation, function, and elimination [29]. Astrocytes have also been suggested to contribute to degeneration in AD and potentially play an important role in the inflammatory profile observed in AD pathology [27]. Upon exposure to toxic materials or injury, astrocytes become activated, transforming both their morphology and function to become so-called “reactive” astrocytes. Much like microglia, two different states of reactive astrocytes have been proposed, namely, “A1” and “A2,” depending on the stimuli. The A1 phenotype has been observed to be neurotoxic, whereas A2 astrocytes possess neuroprotective properties. The former predominates in AD conditions. Astrogliosis, with an increase in reactive astrocytes, has been observed in AD, and this reactivity is especially prevalent around A*β* plaques. Astrocytes have thus been suggested to be activated by A*β*, leading to overexpression of proinflammatory cytokines such as IL-1*β*, TNF-*α*, and IL-6, in addition to increased formation of ROS and NO. Resulting elevated oxidative stress levels might then initiate neuronal degeneration. It has also been proposed that reactive microglia can induce this A1 state by secreting cytokines, further promoting formation of reactive astrocytes and neuroinflammation [27, 30, 31]. Microglia and astrocytes can thus both play beneficial or detrimental roles in the CNS, whereby A*β* accumulation and inability to resolve plaque formation can lead to a chronic neuroinflammatory state as AD progresses, further exacerbating neurodegeneration (Figure 1).

Initiation of the immune response is triggered by recognition of various pathogens and stimuli, and immune cells are able to respond to different infections, trauma, brain injury, protein aggregation, and neuronal death. Upon damage, immune cells migrate to the injury site and initiate an immune response. Microglia and astrocytes are able to recognize such stimuli owing to their expression of specific receptors, called pattern recognition receptors (PRR). These receptors can bind and respond to pathogen-associated molecular patterns (PAMPs) or danger-associated molecular patterns (DAMPs) such as A*β* and in this manner mediate the inflammatory response. It is through this receptor-complex that they can interact and react to the A*β* accumulation that occurs in AD [7]. Several types of PRRs are present on microglia and astrocytes including scavenger receptors, receptors for advanced glycation end products (RAGE), and toll-like receptors (TLRs), with the latter being implicated in AD pathogenesis. TLRs comprise an important group of PRRs, and various types of these receptors are expressed by microglia and astrocytes. TLRs are also found on neurons and oligodendrocytes, and emerging evidence has suggested involvement of these receptors in AD pathology [7, 32].

## 3. Toll-Like Receptors

Toll-like receptors are membrane receptors that can detect and be activated by the presence of pathogens* via* an extracellular domain, thereby generating an inflammatory response. These crucial components of the innate immune system were initially discovered on cells such as macrophages and dendritic cells [58, 59]. Subsequently, TLRs have been identified in a plethora of tissue and cell types including fibroblasts [60], eye tissue [61], blood cells [62] and, of specific interest for this review, brain tissue [63].

So far, TLR 1-13 have been identified in mice with the exception of TLR 10 [64] whereas 10 types of TLRs have been identified in humans (TLR 1-10) [65–68]. While not immediately appreciable, this difference is in fact notable and will be elaborated upon later in this review.

Common to all types of TLRs is their activation by the presence of a microorganism. Since types of microorganisms far exceed that of TLRs, the TLRs do not recognize a specific microorganism but instead recognize common pathogens expressed by different classes of microorganism. These are referred to as pathogen-associated molecular patterns (PAMPs). In addition to recognition of PAMPs, TLRs can interact with endogenous molecules such as proteins, polysaccharides, proteoglycans, nucleic acids, and other cellular components that are released from dead cells or damaged tissues [69]. These components are commonly known as damage-associated molecule patterns (DAMPs) and can also be released upon injury or during stress as an indicator of damage [70].

This section will give a general overview of TLRs with regard to their activation and signaling pathways. This overview will be based upon activation by pathogens, whereas more comprehensive details of activation by specific DAMPs associated with AD will be provided by other sections of this review.

TLRs can be segregated into two groups known as the cell surface TLRs and intracellular TLRs. Cell surface TLRs include TLR 1, 2, 4, 5, 6, and 10 and they can recognize various membrane components from bacteria such as proteins, lipids, and lipoproteins. TLR 3, 7, 8, and 9 are intracellular TLRs that are primarily located in the endosome and lysosome, where they can recognize various forms of RNA and DNA from viruses [71].

**Figure 2:**
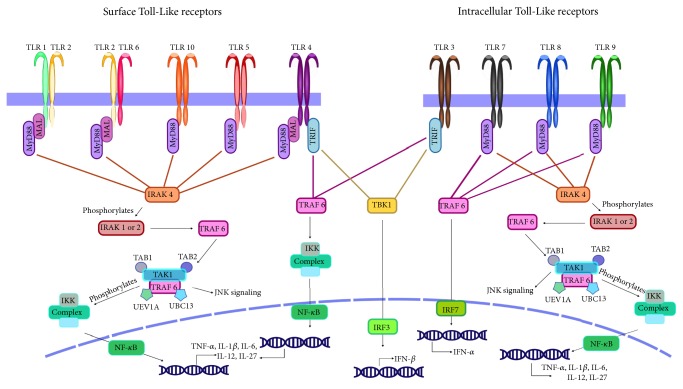
TLR signaling pathways. Depending on which TLR is stimulated, adaptor proteins MyD88, TRAM, TRIF, or MAL will associate with the TIR-site of the receptor. For the MyD88-dependent pathway, MyD88 recruits phosphorylated IRAK1 or 2 and associates with TRAF6. TRAF6 forms a complex with TAB1, TAB2, TAK1, UEV1A, and UBC13. The complex formation activates TAK1 which then phosphorylates the IKK complex. Once phosphorylated it can activate transcription factors involved in JNK signaling and NF-*κ*B which results in production of various proinflammatory cytokines. The MyD88-independent pathway is initiated by TLR3 or 4 where TRIF associates and recruits TRAF6 of TBK1. TRAF6 results in NF-*κ*B activation and TB1 in activation of the transcription factor IRK3, producing IFN*β*. TRAF6 activation can also lead to IFN*α* production if activated by TLR 7, 8, or 9.

TLRs are activated by their respective ligand(s) (Table 1) binding to a leucine-rich repeat motif located on the outside of the membrane. The leucine repeats form a horseshoe structure which helps the ligand to attach to the TLR [72]. After attachment, the TLR will recruit specific adaptor molecules* via* its cytoplasmic Toll/IL-1 receptor (TIR) domain. Adaptor molecules that associate with the TIR-domain include MyD88, MAL, TRAM, and TRIF [73]. Depending on which adaptor molecule is recruited to the TIR-domain, various signaling pathways will be initiated (Figure 2). As an example, if TLR4 is stimulated by the presence of lipopolysaccharide (LPS), it will recruit the MyD88 adaptor molecule to its TIR-domain. MyD88 then associates with interleukin-1 receptor-associated kinase 4 (IRAK4) and IRAK1, forming an active complex that can add a phosphate group to the TNF receptor-associated factor 6 (TRAF6), allowing TRAF6 to form a complex that can phosphorylate the IKK-complex. The IKK-complex is responsible for recruitment of the transcription factor NF-*κ*B to the nucleus where it increases expression of cytokines to mediate an inflammatory response [71, 74]. Studies have shown that, in order for TLR 4 to produce an inflammatory response to LPS, the cofactor CD14 is needed, as no production is seen in its absence [75]. The release of cytokines and other inflammatory factors, caused by TLR stimulation, can initiate a response in surrounding cells, thereby amplifying immune response. The activation of TLRs often results in an upregulation of TLR expression, allowing the cells to detect pathogens more efficiently, producing a stronger inflammatory response due to this positive feedback loop [76].

TLRs function as dimers with different types of TLR receptors forming heterodimers, so increasing ligand diversity. A high diversity of receptors and pathways allows for a highly tailored biological response according to the specific stimulus.

### 3.1. Toll-Like Receptors in the Human Brain

As elaborated upon in the previous section, TLRs respond not only to pathogens but also to the presence of DAMPs. Two main routes of TLR activation occur in neurodegenerative diseases: (1) cells undergoing apoptosis and necrosis release their cellular contents including DAMPs, triggering the immune response interacting with TLRs [77] and (2) other types of inflammation factors and protein aggregates directly activate TLRs [78]. Responses to DAMPs are of specific interest when studying neuroinflammation in the brain, since these are triggers from dying neurons and astrocytes and not caused by bacterial infections. Currently, the stimulation of TLRs* via* DAMPs is poorly studied: most investigations of TLR responses are still performed by presenting pathogen components to elicit an immune response. This following section will summarize the various types of TLRs identified in cells of the human brain, their ligands, and the downstream activating response.

**Figure 3:**
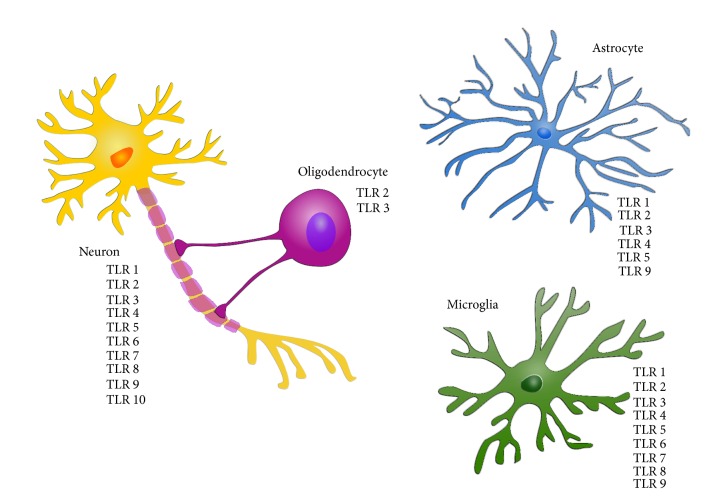
Expression of TLRs in human brain cells. Neurons express all ten human TLRs identified to date while microglia express nine of them. Astrocytes express fewer varieties of TLR and oligodendrocytes only express TLR 2 and TLR 3.

All ten types of human TLRs have been found to be expressed in cells of the human brain [79] (Figure 3). It should be noted that many studies have investigated the expression of TLRs in mouse-derived tissue and cells. However, since TLRs are incompletely conserved between mouse and human, only mRNA and protein encoding TLRs, found in human brain tissue and cells, will be presented in this section.

#### 3.1.1. Microglia

Microglia cells have been shown to express mRNA and protein for nine of the 10 TLRs identified in cells of the human brain (TLR 1-9) [80, 81]. This broad expression profile is not surprising given that microglia comprise the brain's innate immune system and that some of the inflammatory mediators that microglia produce are known to be regulated by TLRs. TLR 1 does not appear to be present in microglia as a homodimer but has been shown to form a heterodimer with TLR 2, responding to the spirochete* Borrelia burgdorferi*, and increases TLR protein and mRNA expression in astrocytes and glial progenitors [6]. This finding is informative in dissecting the pathways underlying neurodegeneration as the* Borrelia burgdorferi* infection in some cases affects the nervous system, leading to dementia [82]. Pathways identified in studies using* Borrelia burgdorferi *might therefore overlap with those involved in DAMP-initiated neurodegeneration.

In other pathological conditions, such as malignant tumors of the glial tissue of the nervous system (glioma), TLR 1/2 heterodimers, together with TLR 2/6 heterodimers and TLR 2 in microglia, facilitate infiltration of gliomas into the brain parenchyma of mice. Interventions into the activation of these TLRs might prevent tumor infiltration, increasing the likelihood of surgical resection [83].

Viral infections such as hepatitis C activate TLR 2 and TLR 6 in human microglia culture. These have been shown to respond to the presence of the hepatitis C virus antigen (virus NS3 protein), releasing the cytokines IL-8, IL-6, TNF-*α*, and IL-1*β* [84].

Another extensive study has systematically investigated the innate immune response mediated by TLRs in human microglia cells [81]. The major findings of this research were that human microglia express mRNA for TLR 1-9. Moreover, microglia could be activated through ligation of TLR 2 with synthetic lipopeptide, TLR 3 with synthetic dsRNA, and TLR 4 with lipopolysaccharide. All of these modes of activation triggered secretion of proinflammatory cytokines such as IL-6, IL-10, IL-12, and TNF-*α*.

All of these studies support the involvement of TLRs in the innate immune response mediated by microglia, as they produce proinflammatory cytokines. This immune response and inflammatory response is intensified by upregulated mRNA and protein expression of TLR 2 and TLR 3 and downregulated mRNA expression of TLR 4 [81]. For this reaction, microglia interact with astrocytes and mediate these responses. These findings underscore that glial activation results in an increased inflammatory response. Persistent activation of inflammatory responses in the glial compartment of the brain is characteristic for neurodegenerative diseases and if homeostasis cannot be restored after the pathogenic components have been removed, these can be considered as potential triggers for disease pathology.

#### 3.1.2. Astrocytes

TLR 2 and TLR 3 are the prevalent TLRs in astrocytes and are both highly expressed on RNA and protein level [80, 85]. For the other TLRs such as TLR 1, 4, 5, and 9, astrocytes have lower expression levels of mRNA [81, 86, 87] and protein [86] while TLR 6, 7, and 8 mRNA and protein are either expressed at very low levels [81, 87] or wholly absent [86]. TLR 2 mRNA has however also been reported to be expressed at negligible levels or not at all in astrocytes [81]. The controversy surrounding levels of TLR astrocyte expression likely reflected differences in detection of TLR between studies. These might stem from astrocytes not being in the same activation status or stimulated in differing manners between studies. This hypothesis is supported by previous work in mice, showing that activation of TLR 2 heterodimers TLR 1/2 and TLR 2/6 in microglia is highly dependent upon the type of stimuli astrocytes have previously been exposed to [39]. Furthermore some use only fetal samples [86, 87], others adult [80], and others again both adult and fetal samples [81]. The culture time for the astrocytes varies from 2 passages [81] to 10 passages [86] which most likely affect the expression level of TLRs. This is supported by a study showing a 212-fold difference in TLR 4 gene expression between astrocytes extracted from human fetal brains and from human adult brains [88].

In regard to activation and response of TLRs in astrocytes, TLR 3 and TLR 4 have received the most attention so far. TLR 3 on human astrocytes has been shown to be activated by exposure to the synthetic compound poly (I:C) resulting in increased production of IL-6, IL-8, and TNF-*α* [85, 86, 89]. The protein expression of TLR 2, TLR 3, and TLR 4 in astrocytes is enhanced if the astrocyte has been activated by proinflammatory cytokines such as IFN-*γ* [80, 83]. This augmented activation by proinflammatory cytokines, mediated through activation of neighboring astrocytes or microglia, has been shown to lead to expression of anti-inflammatory cytokines rendering a neuroprotective effect [83]. Furthermore TLR 3 activation by poly (I:C) has been shown to increase ATP release from lysosomes, stimulating lysosomal clearance of pathogenic substances [90].

TLR 4 can be stimulated by lipopolysaccharides (LPS) from gram-negative bacteria [81] in the presence of CD14 protein [6]. Astrocytes stimulated with LPS increase their expression of TNF-*α*, IL-6, and IL-8 and activate NF-*κ*B [6, 81], all of which are associated with proinflammatory signaling. All of these studies underline the importance of astrocytes within the innate immune response of the brain, closely collaborating with microglia.

#### 3.1.3. Oligodendrocytes

Work on CNS TLRs has mainly focused upon microglia and astrocytes. However, such receptors have also been identified on oligodendrocytes and neurons. Oligodendrocytes are the myelinating cells of the CNS, providing a supporting role for neurons* via* axonal insulation and release of neurotrophic factors. Although little is known in terms of TLR expression and function in oligodendrocytes, mRNA expression of TLR 2 and TLR 3 has been identified in these cells, and activation of these receptors has been suggested to play a role in CNS repair [80]. Besides these findings, an indirect effect of TLR activation* via* activated microglia and astrocytes has been proposed to cause demyelination of oligodendrocytes and their subsequent loss [91]. Therefore, the direct and indirect effects of TLR activation can contribute to degeneration of oligodendrocytes in the brain affecting neurons and their survival.

#### 3.1.4. Neurons

Similar to glial cells, mRNA and protein expression of TLR have been identified in neurons in both the peripheral nervous system and CNS. There has been some controversy in regard to which TLRs are expressed in human neurons. Whilst some studies have identified only some of these, another study has detected all 10 TLRs in human neuronal populations, although the detectable mRNA expression level varied between different neuronal cell types [79]. The neuronal expression of such TLRs allows them to trigger an immune response, indicating the presence of specific neuronal innate immune machinery. The neuronal TLR signaling pathways have been suggested to involve glycogen synthase kinase 3*β* (GSK3*β*), jun-N-terminal kinase (JNK), and phosphatidylinositol 3-kinase/protein kinase B (PI3K/AKT). These factors and pathways have been implicated to play a role in the immune response of the brain as well as being important for brain development and maintenance of brain homeostasis [92].

## 4. Toll-Like Receptors in Alzheimer's Disease

Various cell types and pathways in the human brain display a connection with neurodegeneration. The precise mechanisms causing the neuronal death associated with AD are, however, still unknown. Several studies have implied a role of TLRs in AD pathology, and in this section the potential role of such receptors in AD pathogenesis will be discussed. Our aim is to provide a better understanding of disease mechanisms and the potential of TLRs as druggable targets in future therapeutics. Neuroinflammation and the activation of immune cells are considered a hallmark of AD, and TLRs have been suggested to play a significant role in this activation. Stimulation of TLRs and their response is dependent on the type of stimuli, receptor, and cell population expressing them and, in this section, the AD-specific TLR response will be reviewed.

In comparison to healthy brains, brain samples of AD patients display increased TLR mRNA expression. This tendency has been observed for all TLR groups, with the exception of TLR 2 mRNA [93]. The inflammatory response seen with TLR activation differs depending upon the type of receptor being stimulated and in what combination they are activated. For instance, simultaneous stimulation of TLR 4 and TLR 2, TLR 4 and TLR 9 or TLR 2 in combination with TLR 9 causes a significant increase in inflammation in mouse models [94]. In microglia from mice it has also been observed that inflammation is upregulated if both TLR 1 and TLR 2 are stimulated, compared to a solely TLR 2-mediated response [95]. Silencing of TLRs has been shown to decrease the inflammatory response, further indicating an important role for them in inflammation. This is, however, not seen for TLR 7 in human AD brains, although an upregulation of expression in AD mouse models has been reported [96]. These results indicate TLRs to be associated with noninflammatory processes, and TLR 7 has been suggested to be associated with autophagy in mice [97]. The role of TLRs in AD pathology is therefore very diverse, depending upon the exact receptors involved. However, there are clear indications that these are in fact involved in the neuroinflammation accompanying neurodegeneration. Further studies must be conducted in order to confirm the involvement of TLRs in disease conditions to fully understand the complex signaling mechanisms at play, as a study made on post-mortem brain samples from AD-patients and healthy controls showed a great variation in TLR expression from patient to patient [80]. In addition, some of these findings are based on mouse models and should be confirmed in human models. Animal models do not necessarily recapitulate the precise human disease pathology, also implicit in the divergence in CNS TLR expression between rodents and humans.

An article from 2018 has analyzed the expression profiles from 25 different genetic studies including AD studies [98]. This work has resulted in a public database that includes the changes in expression profile for a gene of interest. For an overview, Table 2 has gathered the results from human TLR 1-10 in AD studies in relation to a healthy control.

As evident from the genetic studies, there are differences between studies of whole tissue and studies of cells, but also differences between human and mouse studies (Table 2). In the following section, all results should be considered carefully, as small differences between studies can cause very different outcomes.

### 4.1. A*β* and Tau in relation to TLRs

The formation of insoluble A*β* plaques and NFTs, the main pathological hallmarks of AD, is suggested to initiate a cascade of pathological events that have been previously reviewed to cause neuronal dysfunction [99]. The involvement of TLRs has been implicated in this cascade: A*β* peptides have been suggested to stimulate TLRs in mice [100] leading to increased mRNA expression of these receptors [93, 101, 102]. Studies in APP mouse models have indicated upregulated levels of mRNAs for TLR 2, TLR 4, TLR 5, TLR 7, and TLR 9, compared to TLR expression in plaque-free tissue. In contrast, TLR 3 mRNA expression was shown not to be significantly altered in AD mouse models, indicating that both activation and response in AD conditions are specific for different types of TLRs [103]. These findings also emphasize the potential differences between TLRs in rodents compared to humans. In contrast to mice, TLR3 mRNA and protein are upregulated in human AD brains and TLR 2 expression is not significantly increased, as previously mentioned. Differences between model organisms should thus be considered in future research, to fully understand the human disease aspect. The increase in TLR expression resulting from A*β* stimulation correlates with increased inflammatory response. For instance, addition of A*β* to mouse hippocampal neurons upregulates TLR 4 protein, which then shows a stronger response to lipopolysaccharide (LPS) treatment, and increased neuronal death [104].

Based on various mouse models, TLRs have also been suggested to play a role in A*β* clearance by microglia, and such phagocytosis is likely dependent upon TLR 2, TLR 4, and TLR 9 [105–107]. TLR 2 mediates interaction between microglia and A*β* and has been suggested to serve as an important trigger for neuroinflammation in AD. Deficiency of TLR 2 in mice has been suggested to reduce inflammation and increase clearance of A*β*, favoring the microglial M2 phenotype and neuroprotection, improving neuronal function. Such deficiency could thus be beneficial by inhibiting A*β*-induced neuroinflammation [95]. In addition, TLR 2 deficiency has been observed to relieve tauopathies in mice, indicating further beneficial effects. These studies imply that TLR 2 activation contributes to inflammation and neurodegeneration, and inhibiting TLR 2 function might potentially slow disease progression. However, there is some controversy regarding TLR 2 and its involvement in AD. Although deficiency of the receptor has been implicated as beneficial, conflicting results have demonstrated TLR 2-mediated A*β* uptake, and activation of TLR 2 with, for instance, peptidoglycan (PGN) has been reported to promote microglial phagocytosis of A*β* in mice. It has been suggested that this promotes M1 microglial activation and a proinflammatory state [108]. The hypothesis that TLR 2 is involved in the proinflammatory microglia response has also been supported by a study showing that the coreceptor CD14 must act together with TLR 2 and TLR 4 in order for fibrillary A*β* to bind and trigger a microglial response in mice [109]. Despite the controversy, these findings clearly indicate a role for TLR 2 in the inflammatory profile associated with AD.

TLR 4 is the other major receptor involved in A*β* activation of microglia. Upregulation of TLR 4 mRNA has been observed in AD transgenic mice, and TLR 4 expression is increased in brain tissue surrounding A*β* plaques [101]. Deficiency of TLR 4 in microglia from such mice has also been demonstrated to increase A*β* deposits [110], indicating that TLR 4 is also required for microglial activation [106].

Besides TLR 2 and TLR 4, the role of TLR 9 in AD pathology and inflammation has been probed by a number of studies. Stimulation of TLR 9 has been demonstrated to increase microglial recognition of A*β*42 [107] and A*β* uptake [110] in mice. TLR 9 can bind DNA containing unmethylated cytosine-guanosine (CpG) sequences, commonly found in bacteria and viruses, and such stimulation has been shown to reduce A*β* in the cortical regions of AD mouse models [111] and restore cognitive function in AD mice as a result of the TLR9 stimulation [112]. This has also been observed in cocultures of neurons and microglia in which stimulation of TLR 9 led to reduced toxicity of oligomeric A*β*, with increased microglial clearance without production of neurotoxic factors [113]. The use of TLR9 agonists in mouse studies has not raised any safety concerns [114], but tests need to be made in humans, as it is likely that the increased inflammatory response, caused by stimulation of TLR9, can have a negative effect even though a study has shown that activation of TLR9 in mice does not worsen A*β*-induced microglial activation [115]. Taken together, these findings render TLR 9 an attractive candidate to investigate further regarding the development of future AD therapies.

Although most studies involving TLRs in AD pathology have focused upon TLR 2, TLR 4, and TLR 9, other TLRs might also potentially play a role in AD development. Interestingly, some genetic variants of TLR 5 in mice have been suggested to be preventive for AD [116]. Expression of the ectodomain of TLR 5, mediated by Adenoviral vectors, has been shown to result in decreased A*β* accumulation. This ectodomain can form a complex with A*β*, thus preventing aggregation and toxicity, making it more susceptible for removal [116]. Studies involving both TLR 9 and TLR 5 thus point towards promising therapeutic potential of TLRs in AD.

Compared to the many studies on A*β* and its interaction with TLRs, limited data is available regarding tau tangles and TLR response. TLR 3 protein expression has been shown to increase correspondingly with the level of tau tangles in human cell culture and brain samples [93]. However, stimulation of TLR 3 did not seem to impact microglial activity in these cases. Conversely, mild stimulation of TLR 4 with LPS in transgenic mice overexpressing human mutant tau in neurons resulted in enhanced autophagy and reduction in phosphorylated tau, indicating that neuroinflammation promotes autophagy. Chronic mild stimulation of TLR 4 might thus possibly attenuate AD-related tauopathy, by providing beneficial neuroinflammation, which might be exploited in AD treatment [117]. These studies hence indicate that TLR signaling might also be linked with tau pathology.

### 4.2. Activation of Microglia

As previously described, A*β* plays a major role in microglial activation in AD. Studies have shown that A*β*42 protofibrils, an intermediate preceding amyloid fibril formation, can trigger the MyD88-dependent pathway in microglia. Such activation favors the M1 microglial phenotype and causes secretion of proinflammatory mediators [118]. Microglial activation can be mediated by TLRs, and expression of TLR 1-9 mRNA is seen in microglial cells. Stimulation of TLR 2, TLR 4, and TLR 9 leads to activation of this cell population, characterized by release of cytokines such as TNF-*α*, IL-1*β*, and IL-12, in addition to nitric oxide. This has been confirmed by* in vitro* studies using agonists for TLR stimulation [119–121], and simultaneous stimulation of microglia with even low concentrations of TLR ligands has been shown to result in an additive effect, indicating that low amounts of pathogens can manifest in TLR activation, if multiple TLRs are targeted [121]. The exact mechanism(s) through which TLR activation can influence AD pathology and if this is the case is not fully understood, as well as the mechanisms causing AD. However, a constituent of the signaling pathways has been identified and is likely mediated by the MyD88/TRAF6//MAPK/IKKs/NF-*κ*B pathway or the MyD88/PI3K/NF-*κ*B pathway, both of which promote M1 activation [122].

Depletion of both TLR 2 and TLR 4 has been observed to decrease microglial activation. However, while deficiency for TLR 2 has been linked with reduced A*β* plaque burden [100], TLR 4 deficiency has been observed to increase A*β* deposition [123]. In contrast, TLR 4 inhibition has been observed to result in reduced secretion of proinflammatory cytokines [101]. The fact that deficiency of these receptors can reduce the inflammatory response in AD suggests an important role of these TLRs in microglial activation. However, activation is not solely dependent upon TLRs, and activation can occur in TLR 2- and TLR 4-deficient conditions* via* other factors such as ROS-mediated activation, but under such circumstances, very high levels of LPS are required to induce an inflammatory response [124].

Suppression of TLR 4 appears to improve cognitive deficits and decreases inflammatory injury in mice with AD mutations [125]. By targeting TLR 4 signaling pathways, inflammation can thus potentially be decreased. Chronic activation of TLR 2 and TLR 4 has been suggested to contribute to neuroinflammation. However, further studies are required as conflicting results remain in terms of the effects of activation and silencing of the same receptors. Conversely, all studies regarding TLR 9 activation of murine microglia have shown consistent results, leading to an ultimate decrease in A*β* [107, 111, 113] as described in the previous section. A*β* has also been observed to induce dimerization of TLR 4 and TLR 6 in mice, and inhibiting this process led to decreased release of proinflammatory cytokines from microglia, providing a neuroprotective effect [126].

Activation of microglia can result in different outcomes, either leading to the proinflammatory M1 state, or the anti-inflammatory M2 state, promoting neurotoxicity and neuroprotection, respectively. The balance between these two phenotypes is essential in terms of neuroinflammation and maintaining brain homeostasis [127]. In terms of neurodegeneration, this balance is shifted towards the M1 microglial phenotype, promoting the release of proinflammatory mediators and neuroinflammation [127]. Whether the observed inflammatory profile in AD is a direct cause of the disease or if it is in fact a secondary reaction to other AD pathologies is, however, still hotly contested.

Some studies have suggested that neuroinflammation in AD occurs due to the fact that microglia become senescent, and thus less responsive to stimuli [128]. Concordantly, repeated treatment of murine microglia with LPS has been demonstrated to drive them towards a senescent state [129]. Mouse studies showing no difference in the prevalence of active microglia between postmortem AD and control brains have supported this theory. The fact that LPS stimulation, which acts on TLR 4, can induce this microglial state invoked the hypothesis that chronic exposure to stimuli such as A*β* leads to less responsive microglia, decreased A*β* clearance, and thus accelerated AD progression [130]. Studies in mice have also indicated that age plays a role in microglial activation. Microglia from older mice have been observed to secrete higher amounts of proinflammatory cytokines compared to those from younger mice [131], and these microglia are less responsive to other stimuli. Chronic activation of microglia can thus lead to a state in which these cells are no longer able to respond to additional stimuli [132].

A recent study has shown that stimulation of microglia can lead to epigenetic reprogramming, traceable for up to 6 months [133]. In this study, two types of immunological imprinting were distinguished from one another, namely, training and tolerance, which can respectively enhance or suppress the inflammation [133]. This finding emphasizes that the type of stimulus can influence the inflammatory signaling pathway and produce distinct outcomes despite targeting the same TLRs.

### 4.3. Reactive Astrocytes

Besides microglia, reactive astrocytes play a role in neuroinflammation and neuronal death in AD. Activation of TLRs in human and rat astrocytes leads to secretion of TNF*α*, IL-6, IL-8, IL-10, IL-1*β*, and inducible nitric oxide synthase [86, 87, 134, 135]. TLR stimulation in these cells likely involves the NF-*κ*B signaling pathway, which has been shown to induce astrogliosis and neuroinflammation in mice [136]. Increased TLR 2 expression in astrocytes has also been demonstrated to increase the secretion of proinflammatory cytokines, further indicating that TLRs are implicated in the inflammatory response. In contrast, astrocytes from TLR 2-deficient mice have been found to show reduced production of inflammatory mediators [137].

Furthermore, activation of TLR 3 in rats has been seen to increase the proinflammatory phenotype of astrocytes, contributing to neurotoxicity [138], whilst TLR 9 stimulation in mice has resulted in reactive astrogliosis, further emphasizing the role of TLRs in neurodegeneration [139].

Astrocytes can thus be activated by TLR recognition of different stimuli. However, these cells can also respond to cytokines of the adaptive immune system such as IFN-*γ* and TNF-*α*. Innate signals such as LPS and TLR ligands have been shown to elicit a stronger upregulation of TLRs and increase in cytokine release compared to cytokine-stimulated astrocytes. These findings clearly indicate that different stages of neurodegeneration can generate altered responses in astrocytes and are important for understanding the role of astrocytes in inflammation and neurodegeneration [140].

### 4.4. Implications of TLR Activation in Neurons

Activation of TLRs can produce either direct or indirect effects on the neuronal population of the CNS. The direct effect of TLRs can be seen from studies of knock-out mice. In TLR 2-deficient mice, differentiation of neural progenitor cells into neurons is favored over astrocytes, resulting in reduced plasticity while* TLR 4*^*-/-*^ mice show increased proliferation and differentiation of neural progenitor cells [141]. Together, these findings show that TLRs are involved in neurogenesis and therefore most likely are involved in neurodegenerative mechanisms of AD. In accordance with this notion, mouse models have shown that neurons can respond directly to the presence of A*β* through TLR 4, and such stimulation can lead to apoptosis [142]. By downregulating TLR 4, neurons showed greater survival and less sensitivity to A*β*. The same study looked at the levels of TLR 4 in brains from AD patients and healthy controls and found lower TLR 4 levels in AD patients, indicating that neurons expressing TLR 4 died. TLRs thereby directly impact neuronal health in AD. Because TLR 4 is also expressed in healthy neurons, apoptosis cannot be explained by the presence of TLR 4 alone but it is clearly involved in the process.

Furthermore, neurons can be affected by the neuroinflammation initiated by microglial activation as this process initiates a cascade of proinflammatory events. Stimulation of TLR 2 and TLR 4 in mice by A*β* activates microglia and causes secretion of proinflammatory cytokines [105] which can have detrimental effects on the surrounding neurons, hence promoting neurodegeneration.

A connection between neurons, neurodegeneration, and TLRs has also been found in human brain samples of patients with Parkinson's disease [143], where expression of the TLR 2 protein was found to be increased in patients. The same study showed that activation of TLR 2 in human cells increased the production of *α*-synuclein, a well-known hallmark of Parkinson's disease, but also a protein that has been associated with AD [144].

The activation of TLRs can thus affect the neuronal population, directly or through microglia-mediated inflammation, both of which should be studied further to increase our understanding of how these pathways work together to exacerbate neurodegeneration.

### 4.5. Aging/Stress

The number of people affected by dementia is expected to reach 152 million by 2050, due predominantly to increased longevity [145].

Many studies have shown that chronic stress increases the risk of developing AD as the body cannot normalize its homeostasis which progressively affects the physiological balance [146], leading to neurodegeneration [147]. Stress in fAD mice has been shown to mainly affect the hippocampal region of female mice, indicating stress pathology to be region- and sex-specific [148].

Chronic stress can lead to induction of proinflammatory mechanisms, causing oxidative stress due to generation of oxidative species [146]. As humans are exposed to stress throughout their lives, it is not a direct cause of AD, but stress might increase the level of damage in brains susceptible to neurodegeneration. It is therefore of interest to study the effect of stress on aging cells, as these are more susceptible to damage [149]. This effect has been studied in neonatal mouse microglia cells cultured for 16 days* in vitro* and investigated on days 2, 10, and 16 [127]. On day 2, microglia showed adaptable morphology and expressed markers of reactive phenotype whereas microglia on day 16 showed branched morphology, increased NF-*κ*B activation, and glutamate release. Thus, old microglia cells (day 16) behave in a similar fashion as irresponsive/senescent microglia. Microglia from old mice secrete greater amounts of IL-6 and TNF-*α* compared to those from young mice and are less responsive to stimulation [131]. These findings indicate a higher detrimental effect of stress in aging microglia, supporting the hypothesis that brains of elderly people are more vulnerable to neuroinflammation.

In relation to TLRs, expression of TLR 2 and TLR 4 in microglia has been shown to decrease with age [104] together with the capacity to migrate and phagocytose. In correlation with this, the general level of functional TLR 1, 6, and 10 in human DNA from healthy old people has been shown to decrease [150], indicating that a downregulation of these TLRs in general might provide a beneficial effect in aging.

Other genetic studies have highlighted the potential influence of TLRs in AD, in which TLR 2 emerges as a potential risk factor in late onset AD [151, 152]. In a genetic study of a Chinese population, TLR 2 was not identified as a significant genetic risk factor for AD [153]. This might be explained due to differences in populations and testing protocols. All these findings together with the observation that mice deficient for TLRs show less cellular damage after exposure to stress [154] confirm that a relation between age, stress, TLRs, and inflammation exists but that further studies are needed to elucidate their relationships to one another.

### 4.6. Components Known to Decrease Inflammation via TLR Pathways

Ever-increasing numbers of studies have investigated potential therapeutics targeting the TLR signaling pathway to decrease neuroinflammation.

Treatment with Picroliv in mouse brains has been demonstrated to reduce the effect of the TLR 4/NF*κ*B pathway, resulting in decreased expression of TLR 4, BDNF, IL-1*β* protein, and A*β* levels [155].

Stachydrine also reduces the levels of IL-1*β*, TNF*α*, and INF-*γ via* the TLR 4/NF*κ*B pathway upon brain injury [156]. Treatment with Betainine and various polyphenols also exhibits anti-inflammatory effects by decreasing production of proinflammatory cytokines and increasing the release of anti-inflammatory cytokines [157, 158]. This shows that Betainine treatment promotes conversion of microglia from the M1 stage to the M2 stage, which is achieved by suppression of the TLR 4/NF*κ*B pathway.

Together, these studies show that targeting the TLR 4/NF-*κ*B-pathway decreases inflammation, rendering this pathway of therapeutic potential. As members of the NF*κ*B family in general regulate inflammation by mediating synthesis of proinflammatory proteins, they are potential druggable targets for decreasing inflammation [159].

As another study has shown that combinatorial TLR activation results in increased inflammatory response and that the response depends on which specific TLRs are activated [94], other pathways and TLRs should be studied further to dissect potential involvement in AD pathogenesis.

## 5. Discussion

### 5.1. Study of TLR in Human versus Mouse

The various studies presented above were conducted in different models: while some pertain to human cells/tissues, the vast majority was performed in rodents. All of the TLRs identified in humans are also expressed in mice. However, the mouse exhibits three additional TLR members not found in humans [71].

While numbers of TLR members expressed between mouse and human brain cells diverge, so too do the expression* levels* of each member. Mouse astrocytes express TLR 1-6 and very low levels of TLR 7-9 [140, 160] whereas human astrocytes only express TLR 1-6 and 9. In neurons, humans express all 10 TLRs, whereas studies in mice have shown their cortical neurons to only express TLR 2, 3, and 4 [161]. Other significant differences between the innate immune systems of mouse and human include the finding that RNA is sensed by TLR 3, 7, and 8 in humans but by TLR 13 in mice, a receptor that does not exist in human cells [71, 162]. Furthermore, human TLR 9 recognizes the GTCGTT DNA sequence from bacteria whereas mouse TLR 9 recognizes the GACGTT sequence [163]. These studies clearly reveal substantial differences in numbers, expression levels, and cell type-specific expression patterns between mouse and human which need to be taken into consideration if mouse models are employed to study the role of TLRs in neurodegenerative diseases and to identify potential drugable targets.

Despite these differences, mice remain the most common model to investigate AD and other human diseases. Mice are important* in vivo* models since they can easily be bred, and knock-out, transgenic, and knock-in lines have been generated for diverse studies. Furthermore, humans and mice share many genetic and physiological similarities, which have helped elucidate many pathways in mice, which have then subsequently been confirmed in humans. [164]. However, major disadvantages of mouse models are that mice do not naturally develop AD and their longevity is too brief to develop the hallmarks of sporadic AD [165]. Therefore, in order to investigate AD pathology in mice, either transgenic mouse models with several strong pathogenic mutations are employed [166], or some of the pathogenic hallmarks such as A*β* or tau are directly injected into the mouse brain [167–169].

Taken together, owing to the challenges of TLR divergence between mouse and man and the difficulty in recapitulating AD pathology in mouse models, alternative experimental models should be sought.

### 5.2. Use of iPSC Models and Future Studies

One potential model for studying the functional roles of TLR in AD is the use of induced pluripotent stem cells (iPSC). iPSC possess the advantage that they can easily be generated from human fibroblasts [170] collected from skin samples, blood, or even urinary epithelial cells. This allows for investigation in cell lines generated from different individuals and thereby cell lines with different genetic backgrounds. Comparative studies can be made as samples can be taken from both AD patients and healthy controls.

Furthermore, gene-editing technologies such as TALENS and CRISPR-Cas9 allow for insertion of pathogenic mutations into healthy control iPSC or for correction of pathogenic mutations in patient iPSC, allowing for the establishment of isogenic control lines with the same genetic background. Many protocols have been developed to differentiate iPSC into various cell types, such as astrocytes [171, 172], neurons [173], and microglia [174]. It will be very interesting to investigate the expression patterns of TLRs in iPSC-derived neurons, astrocytes, microglia, and oligodendrocytes and to compare these with human brain samples in order to validate these* in vitro* models.

If the same expression patterns in the diverse iPSC-derived models can be validated, these cells would represent valuable tools for the identification of compounds to develop drugs targeting TLR activity and innate immune responses as well as for understanding the human-specific function of TLRs. Another possibility in order to study the TLR responses of human-derived cells in a complex* in vivo* system would be the transplantation of such cells into humanized AD mouse models, even though the investigation of such transplants is hindered by the fact that these mice must remain in an immunocompromised state.

## 6. Conclusion

In this review we have presented the different TLR expression patterns in the main cell types of human brains, their responses to pathogenic triggers, and secretion of proinflammatory cytokines. These different cell types are closely dependent on the innate immune responses of each other and facilitate either increased immune responses or restoration of the homeostatic state depending on the environmental situation in the brain. Moreover, we have described and discussed that microglia and astrocytes specifically respond to A*β* and tau, underlining the importance of TLR-mediated innate immune response in AD. Since the responses to A*β* and tau are late pathological events, the responses to DAMPs released by degenerating neurons are even more intriguing in order to understand early AD pathology linked to inappropriate innate immune responses and potential drug development targeting the mild cognitive impairment state of the disease. Moreover, we have discussed the divergence in numbers and expression patterns of human- and mouse-specific TLRs in the brain, emphasizing the importance of human* in vitro* models, such as iPSC, to investigate the human-specific innate immune response in the various brain cell types facilitated by TLRs.

In conclusion, more studies are needed to elucidate the impact of TLRs in the human-specific context and in relation to AD.

## Figures and Tables

**Table 1:** Overview of Toll-like receptors and their binding ligands.

Receptor	Ligand *(origin)*
TLR 2	Lipopeptides (*surface of gram positive bacteria*)[33], peptidoglycan (*surface of gram positive bacteria*)[34], Zymosan (*surface ligand on Fungi*)[35], Neisserial porins (*gram negative bacteria*)[36]

TLR 1/TLR 2	Triacylated lipopeptide (*surface of gram positive bacteria*)[37]

TLR 2/TLR 6	Diacylated lipopeptide (*surface of gram positive bacteria*)[38], FSL-1 (*synthetic lipopeptide derived from Mycoplasma salivarium*) [39], High mobility group box 1 protein (HMGB1) (endogenous *DNA-binding protein*) [40]

TLR 3	Polyinosinic:polycytidylic acid (Poly(I:C)) (*synthetic ligand with similar structure to dsRNA*)[41], genomic RNA and dsRNA (*Viral RNA*)[42], Stathmin (*endogenous human protein*) [43]

TLR 4	Lipopolysaccharide (LPS) (*molecule isolated from cell membrane of gram negative bacteria*)[44, 45], Glycosylphosphatidylinositol (GPI) (*membrane anchors in Protists*)[46], High mobility group box 1 protein (HMGB1) (endogenous *DNA-binding protein*) [40]

TLR 4/TLR 6	Amyloid beta (*peptides derived from the amyloid precursor gene*)[47]

TLR 5	Flagellin (*structural part of the flagella found on various bacteria*)[48]

TLR 7	ssRNA (*virus*)[49], Imidazoquinoline derivatives (*anti-viral organic compound*)[50]

TLR 8	ssRNA (*virus*)[51]

TLR 9	DNA (*virus *[52]*, fungi *[53]*, protists *[54]* and gram positive *[55]* and negative bacteria *[56]), CpG oligodeoxynucleotides (synthetic single stranded DNA molecules) [57]

TLR 10	Unknown

**Table 2:** Overview of TLR expression in various AD or LPS studies compared to a healthy control. Data from database (http://research-pub.gene.com/BrainMyeloidLandscape).

Receptor	Mouse	Mouse	Human	LPS treatment	
	cell studies	Whole tissue	Whole tissue	^*1*^ *Microglia* ^*2*^ *Cortical*	
TLR 1	Up	Up	Not significant	^1^Up	^2^Up
TLR 2	Up	Up	Up	^1^Up	^2^Up
TLR 3	Down	Up	Up	^1^Down	^2^Up
TLR 4	Down	Up	Up	^1^Down	^2^Down
TLR 5	Down	Up	Up	^1^Down	^2^Down
TLR 6	Down	Up	Up	^1^Up	^2^Up
TLR 7	Not significant	Up	Up	^1^Down	^2^Up
TLR 8	Not significant	Not significant	Up	^1^Up	^2^Up
TLR 9	Down	Up	Up	^1^Up	^2^Same
TLR 10	Not tested	Not tested	Up	Not tested	
